# Application of Gaussia luciferase in bicistronic and non-conventional secretion reporter constructs

**DOI:** 10.1186/1471-2091-15-14

**Published:** 2014-07-09

**Authors:** Christin Luft, Jamie Freeman, David Elliott, Nadia Al-Tamimi, Janos Kriston-Vizi, Jacob Heintze, Ida Lindenschmidt, Brian Seed, Robin Ketteler

**Affiliations:** 1 Medical Research Council, Laboratory for Moleclar and Cell Biology, University College London, Gower Street, London WC1E 6BT, UK; 2 Massachusetts General Hospital, Center for Computational and Integrative Biology, Boston MA02114, USA

**Keywords:** Non-conventional secretion, Gaussia luciferase, GGA1, Bicistronic expression, Signal peptide

## Abstract

**Background:**

Secreted luciferases are highly useful bioluminescent reporters for cell-based assays and drug discovery. A variety of secreted luciferases from marine organisms have been described that harbor an N-terminal signal peptide for release along the classical secretory pathway. Here, we have characterized the secretion of Gaussia luciferase in more detail.

**Results:**

We describe three basic mechanisms by which GLUC can be released from cells: first, classical secretion by virtue of the N-terminal signal peptide; second, internal signal peptide-mediated secretion and third, non-conventional secretion in the absence of an N-terminal signal peptide. Non-conventional release of dNGLUC is not stress-induced, does not require autophagy and can be enhanced by growth factor stimulation. Furthermore, we have identified the golgi-associated, gamma adaptin ear containing, ARF binding protein 1 (GGA1) as a suppressor of release of dNGLUC.

**Conclusions:**

Due to its secretion via multiple secretion pathways GLUC can find multiple applications as a research tool to study classical and non-conventional secretion. As GLUC can also be released from a reporter construct by internal signal peptide-mediated secretion it can be incorporated in a novel bicistronic secretion system.

## Background

Secreted luciferases are versatile tools to investigate cell-based processes based on their high sensitivity, lack of toxicity, small size, wide dynamic range for quantitation and potential for multiplexing. The first secreted luciferase identified was isolated from the marine ostracod crustacean, *Cypridina hilgendorfii*, now named *Vargula hilgendorfii*[1]. Later, secreted luciferases were identified in the marine copepod crustaceans *Gaussia princeps*[2], *Metridia longa*[3], *Metridia pacifica*[4] and, more recently, from copepods of the *Heterorhabdidae, Lucicutiidae* and *Augaptiloidae* families [5,6]. With the exception of *Vargula luciferase,* that uses luciferin, all secreted luciferases utilize the substrate coelenterazine to convert light into a bioluminescent signal. Recently, a small, secreted luciferase termed Nanoluc engineered from a deep-sea shrimp luciferase has been developed that utilizes furimazine as substrate in an ATP-independent reaction [7].

One hallmark of secreted luciferases is their flash-type kinetics which has limited the use in drug discovery high-throughput screening applications. This hurdle has been overcome by engineering of *Gaussia luciferase* with glow-type characteristics [8-10]. *Gaussia luciferase* has been used to measure various cellular processes in cell-based assays as well as *in vivo*[11]. A split-GLUC system has been developed [12,13] and used for imaging of receptor-ligand interactions [14], virus-host protein interactions [15] and oligomer formation of amyloid beta peptide [16]. A highly useful property of *Gaussia luciferase* is its stability in body fluids such as blood and urine. This has facilitated the development of *in vivo* sensors for analysis of tumour growth [17] and caspase activation [18], among others. Secretion of *Gaussia luciferase* is mediated by a conventional N-terminal signal peptide. Inducible release of *Gaussia luciferase* from an intracellularly retained version is possible [19], facilitating the development of intracellular biosensors. Two modifications are necessary for the development of such “luciferase release assays”: one is the deletion of the N-terminal signal peptide (dNGLUC) and the second is the attachment of an anchor to dNGLUC, either by linkage to the actin cytoskeleton or other intracellular retained proteins [18,19]. The first luciferase release assay was used to monitor caspase 8 and 9 activation [19] and autophagy protease ATG4B activation [20]. Later, biosensors for caspase 3 [21], HCV protease [22] and caspase 1 [18] have been developed, all based on the proteolytic cleavage of a peptide sequence that is inserted between *Gaussia luciferase* and the anchor molecule, thus resulting in liberation and export of the luciferase from cells. The non-invasive nature of the luciferase release assay makes it a good assay for high-throughput screening and drug discovery applications and has been successfully used to identify molecular regulators of apoptosis and autophagy [20,23-26]. However the mechanism of the non-conventional secretion that underlies the luciferase release assay is poorly understood. In this study, we have identified molecular determinants that regulate non-conventional secretion of dNGLUC and have studied the role of autophagy in this process.

## Results

### Non-conventional release of Gaussia luciferase

**Figure 1:**
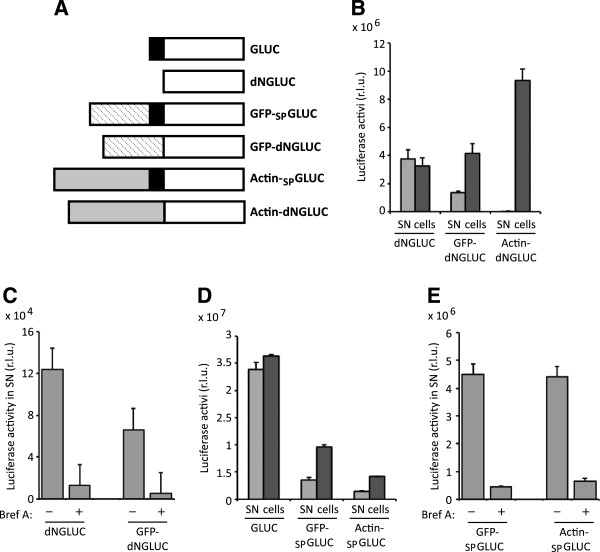
**Three types of secretion for Gaussia luciferase activity. A**, Constructs used in this study are wild-type Gaussia luciferase (GLUC), an N-terminal deletion mutant that has no signal peptide (dNGLUC), GFP-tagged GLUC with an internal signal peptide (GFP-_SP_GLUC), GFP-tagged dNGLUC and β-actin-tagged GLUC with and without an internal signal peptide (Actin-_SP_GLUC, Actin-dNGLUC). The signal peptide is shown as a black box, GLUC without signal peptide is shown in white, GFP as a striped box and β-actin as a grey box. **B**, 293 T cells were transfected with dNGLUC, GFP-dNGLUC and β-actin-dNGLUC, supernatants and cell lysates were collected after 24 h and analyzed for luciferase activity. GLUC activity was measured as described in Methods. **C**, 293 T cells were transfected with dNGLUC and GFP-dNGLUC and cultured for 24 h prior to a media change. Fresh medium containing DMSO or 10 μg/mL Brefeldin A was added to the cells and supernatants were collected after 2 h for analysis of luciferase activity. **D**, 293 T cells were transfected with GLUC, GFP-_SP_GLUC and β-actin-_SP_GLUC and analyzed for luciferase activity in supernatants after 24 h. **E**, 293 T cells were transfected with GFP-_SP_GLUC and βActin-_SP_GLUC, treated with Brefeldin A and supernatants were collected and analyzed for luciferase activity as described. B-D, Shown is the mean of three independent experiments with standard error bars. R.L.U. – relative light units, SN – supernatant.

This study examines the suitability of wild-type GLUC and dNGLUC, a deletion mutant without N-terminal signal peptide, as reporter systems to study protein secretion. Multiple tagged versions of GLUC and dNGLUC were generated as shown in Figure 1A. These include green fluorescent protein (GFP)-tagged versions of GLUC and dNGLUC as well as β - actin tagged versions. First, it was confirmed that dNGLUC is efficiently released from cells in the absence of a signal peptide, whereas β-actin-tagged dNGLUC is retained inside cells (Figure 1B). One possible explanation is that dNGLUC harbors a new N-terminal secretion signal that would allow export from the cells. In order to test this hypothesis, dNGLUC was tagged with GFP to generate GFP-dNGLUC. GFP-dNGLUC was released from cells to a similar extent as dNGLUC and release for both dNGLUC and GFP-dNGLUC was sensitive to treatment with Brefeldin A, thus suggesting that ER-Golgi trafficking is required (Figure 1B,C).

GFP- and β-actin-tagged versions of wild-type GLUC with an internal signal peptide between the two open reading frames as well as wt GLUC were also tested for release of luciferase activity from cells. As expected, wild-type GLUC was released at high levels from cells. In addition, GFP-tagged GLUC and β-actin-tagged GLUC were also secreted at moderate levels from cells (Figure 1D), despite the absence of an N-terminal signal peptide. Secretion of GFP-_SP_GLUC and β-actin-_SP_GLUC was sensitive to treatment with Brefeldin A (Figure 1E). Unlike β-actin-dNGLUC without internal signal peptide, β-actin-_SP_GLUC with internal signal peptide was not retained in cells. One hypothesis is that an internal signal peptide is sufficient to mediate release of the downstream peptide sequence.

### Bicistronic reporter gene expression

**Figure 2:**
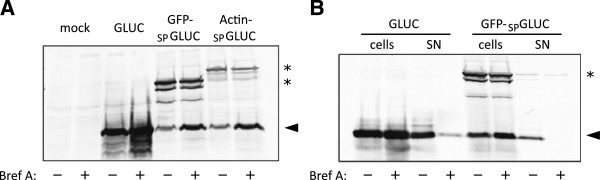
**Bicistronic expression system for release of Gaussia luciferase activity. A**, whole cell lysates from transfected 293 T cells described in Figure 1E were subjected to 10% PAGE and subsequent immunoblotting using a rabbit anti-GLUC antibody. Bands corresponding to GLUC are indicated by an arrowhead and bands corresponding to intact GFP-_SP_GLUC and β-actin-_SP_GLUC are indicated by asterisks. **B**, 293 T cells transfected with GLUC or GFP-_SP_GLUC were analyzed for GLUC activity in cell lysates and supernatants by immuno-blotting with anti-GLUC antibody. Supernatants and cell lysates were collected from the same well. Supernatants were enriched as described in Methods. Cell lysates and supernatants were resolved by PAGE and bands were detected with anti-GLUC antibody. Bands corresponding to GLUC are indicated by an arrowhead and bands corresponding to intact GFP-_SP_GLUC are indicated by an asterisk. Experiments have been performed three times and shown are representative blots. SN – supernatant.

It has been shown before that internal signal peptides have the potential to result in secretion of the downstream gene and can be cleaved by signal peptidase [27]. To examine whether the internal signal peptide in GFP-_SP_GLUC is cleavable, we performed immuno-blotting using a polyclonal antibody raised against the C-terminal part of GLUC. In untreated 293 T cells transfected with GLUC, we observed an expected band at ~20 kDa that increased in intensity upon treatment with Brefeldin A, consistent with accumulation of GLUC inside cells upon blockage of release (Figure 2A). For GFP-_SP_GLUC and β-actin-_SP_GLUC multiple bands were observed including a band at 47 kDa and 62 kDa, respectively, that corresponds to the length of the full-length protein, as well as a band at 20 kDa that corresponds in size to wild-type GLUC. Upon treatment with Brefeldin A, the 20 kDa band increased in intensity, while the bands corresponding to full-length GFP-_SP_GLUC or β-actin-_SP_GLUC did not change in intensity. Therefore, we proposed that GFP-_SP_GLUC and β-actin-_SP_GLUC are cleaved at the site of the signal peptide and the cleaved GLUC but not the full-length construct is released from cells. To test this hypothesis, we enriched supernatants from serum-free media and performed immuno-blotting of cell lysates and enriched supernatants in the presence or absence of Brefeldin A (Figure 2B). From cells transfected with GLUC, a strong band at ~20 kDa was observed in supernatants that was reduced upon treatment with Brefeldin A. Similarly, from cells transfected with GFP-_SP_GLUC, a single band at ~20 kDa was observed that was significantly reduced in supernatants and increased in cell lysates upon treatment with Brefeldin A (Figure 2B). No band corresponding to full length GFP-_SP_GLUC was observed in the supernatant, indicating the protein needs to be cleaved for secretion. We therefore conclude that the strong GFP-_SP_GLUC secretion is mediated by cleavage of an internal signal peptide.

### GLUC is heat-stable

**Figure 3:**
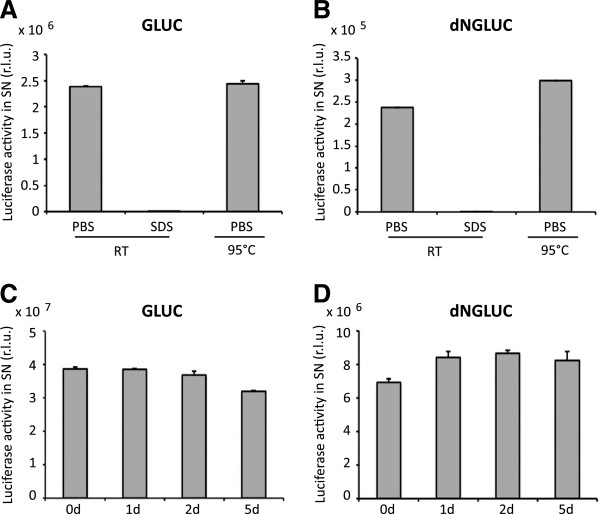
**GLUC and dNGLUC activities are heat-stable. A**, **B**, 293 T cells were transfected with GLUC or dNGLUC and supernatants were collected after 24 h. 10 μL of supernatant were mixed in 90 μL of PBS, or PBS/1% SDS at room temperature or in 90 μl PBS incubated at 95°C for 5 min before analysis. **C**, **D** Supernatants from cells expressing GLUC or dNGLUC were incubated at 37°C for the indicated time points prior to analysis in a plate reader. A-D, shown is the mean plus standard deviations of three experiments. R.L.U. – relative light units, SN - supernatant.

We noted that GLUC activity in supernatants was very stable and that the supernatants could be stored at room temperature for several days without apparent loss of luciferase activity. GLUC and dNGLUC activities were readily detectable when supernatants were mixed with PBS, and were absent following treatment with 1% SDS (Figure 3A,B). Incubation of supernatants at 95°C for 5 min did not result in a significant reduction of GLUC activity (Figure 3A,B). In order to test whether GLUC and dNGLUC are stable at elevated temperatures, thus facilitating the use in time kinetic measurements at physiological temperatures, the half-life of GLUC and dNGLUC at 37°C was determined in a timecourse experiment (Figure 3C,D). GLUC and dNGLUC activity did not show any loss of activity even after incubation for 5 days at 37°C. Thus, GLUC and dNGLUC are very stable reporter proteins that can be used for kinetic measurements of protein expression and secretion at physiological temperatures.

### Non-conventional GLUC release is not stress-induced and does not require autophagy

**Figure 4:**
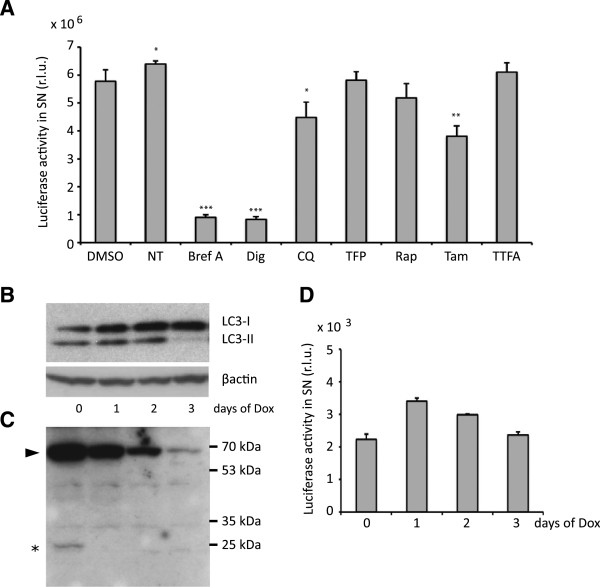
**Release of Gaussia luciferase does not require autophagy. A**, 293 T cells were treated with 10 μM of digitoxin (Dig), chloroquine (CQ), Trifluorperazine (TFP), rapamycin (Rap), tamoxifen (Tam), or theonyltrifluoracetone (TTFA), 10 μg/mL Brefeldin A, DMSO or left untreated (NT) and analyzed for luciferase activity in supernatants (*P < 0.05; **P < 0.01; ***P < 0.001). **B**, embryonic fibroblasts from ATG5 inducible knockout mice were transduced with dNGLUC. ATG5 knockout was induced using 1 μg/mL of doxycycline. Cells and supernatants were collected on three consecutive days after induction. Cells were washed and incubated in fresh medium for an additional 2 h before collection of supernatant. The efficiency of ATG5 knockout was verified by immunoblotting with detetction of LC3 **(B)**, β-actin, and ATG5 **(C)**. **D**, Luciferase activity in the correspondent supernatants was measured using the Perkin Elmer Envision II at the indicated time points. A, B, shown is the mean plus standard deviation of three experiments. Significances were calculated with a two-sided paired t-test. R.L.U. – relative light units, SN - supernatant.

Recently, it has been observed that autophagy can contribute to secretion of cytoplasmic proteins by a non-conventional secretion route [28-31]. In order to determine whether autophagy is involved in the release of dNGLUC, 293 T cells were treated with various agents that are known to induce or inhibit autophagy (Table 1, Figure 4A), as well as Brefeldin A and digitoxin as autophagy independent controls. Inducers of autophagy such as rapamycin did not increase secretion of dNGLUC. Blocking autophago-lysosomal fusion by treatment with chloroquine had only a minor effect on release of dNGLUC from cells (Figure 4A). None of the treatments except digitoxin had a significant effect on overall cell viability (Table 1), thus supporting the notion that neither induction nor inhibition of autophagosomal processes has an effect on dNGLUC release from cells.

**Table 1:** Effect of autophagy modulators on dNGLUC secretion

**Autophagy regulator**	**Mode of action**	**dNGLUC activity in sn in r.l.u (×10**^**5**^**)**	**Viability (norm. to DMSO)**
Controls			
DMSO		57.8 ± 4.1	1.0
No treatment		63.9 ± 1.1	1.0
Brefeldin A	Inhibition of Golgi trafficking [32]	9.1 ± 0.9	1.0
Digitoxin	Inhibition of sodium-potassium ATPase [33]	8.4 ± 0.9	0.4
Autophagy inhibitors			
Chloroquine	Prevention of lysosome acidification and lysosome autophagosome fusion [34]	44.8 ± 5.5	0.7
Autophagy activators			
Trifluoperazine	Dopamine antagonist [34]	58.1 ± 3.1	1.2
Rapamycin	Inhibition of mTOR [35]	51.8 ± 5.1	0.8
Tamoxifen	Inhibition of mTOR signaling via increased ceramide levels [36,37]	38.1 ± 3.6	0.8
Thenoyltrifluoro- actetone	Inhibition of mitochondrial electron transport [38]	61.1 ± 3.3	0.9

In order to test whether cells defective in autophagy would exhibit dNGLUC release, murine embryonic fibroblasts from an inducible ATG5 knockout mouse [39] were transduced with dNGLUC and treated with doxycycline for up to 3 days. ATG5 is essential for LC3 lipidation and the formation of the autophagic membrane. After 3 days of treatment, autophagy was defective in these cells as indicated by an absence of the lipidated form of LC3, LC3-II (Figure 4B) and an absence of ATG5 (Figure 4C). Release of dNGLUC from autophagy-defective cells was not decreased (Figure 4D), indicating that autophagy is not required for the release of dNGLUC.

### Release of Gaussia luciferase can be enhanced by stimulation with growth factors

**Figure 5:**
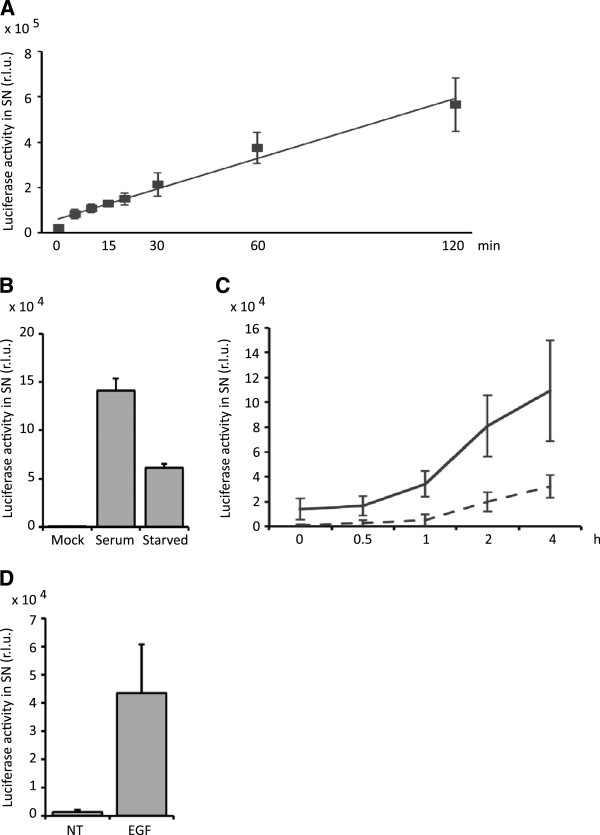
**Secretion of dNGLUC is induced by growth factor stimulation. A**, 293 T cells were transfected with dNGLUC and cultured overnight. Cells were washed once in PBS and fresh media was added. Supernatants were collected at the indicated time points prior to analysis on a PerkinElmer Envision multilabel plate reader. **B**, C, 293 T cells expressing dNGLUC were cultured for 24 h and then starved in 0.1% FCS overnight. Media containing serum (full line) or no serum (dotted line) was added back to the cells and luciferase activity in supernatants was measured after 4 h **(B)** or at the indicated time points **(C)** using a PerkinElmer Envision II plate reader. Shown is the mean of 5 independent transfections with standard deviation. **D**, COS-7 cells were transiently transfected with pEAK12-dNGLUC using calcium phosphate precipitation, cultured for 24 h and then starved overnight in 0.1% FCS. Cells were then stimulated with 100 ng/mL EGF at 37°C for 2 h and supernatants were harvested. **C**-**D**, shown is the mean of three experiments plus standard deviations. R.L.U. – relative light units, SN - supernatant.

In order to assess whether Gaussia luciferase can be used for kinetic measurements of rapid cellular processes, the kinetics of dNGLUC release from cells were determined. The amount of dNGLUC in cell supernatants at various time points after a PBS wash and media change was measured. dNGLUC is a very sensitive reporter gene and release into supernatants was detected as early as 5 min after media change (Figure 5A). The amount of luciferase activity in supernatants increased in a linear manner over time up to 2 h, indicating a steady-state release into supernatants. No sign of cell death or damage was observed, even at later time points, that might explain release. In contrast, treatment with cytotoxic agents typically reduced the amount of dNGLUC detected in supernatants, suggesting that dNGLUC release requires cell vitality (see Figure 4A).In order to test whether release of dNGLUC from cells could be controlled by growth-promoting factors, luciferase release from cells expressing dNGLUC in serum-free media were compared to cells in complete media. A significant reduction in dNGLUC release was observed compared to that of cells cultured in complete media (Figure 5B). Next, the kinetics of release of dNGLUC in serum-free and serum-containing media was explored. As can be seen in Figure 5C, serum strongly enhanced release of dNGLUC within 1 h of addition to the culture, indicating an early response to growth factors. To determine whether this effect could be attributable to growth factors as opposed to other serum constituents, COS-7 cells expressing dNGLUC were treated with 100 ng/ml EGF for 2 h and release of dNGLUC into supernatants was analyzed. Epidermal Growth Factor alone strongly stimulated release of dNGLUC into supernatants (Figure 5D), indicating that release of dNGLUC is responsive to growth factor treatment.

### Non-conventional GLUC release is suppressed by GGA1

**Figure 6:**
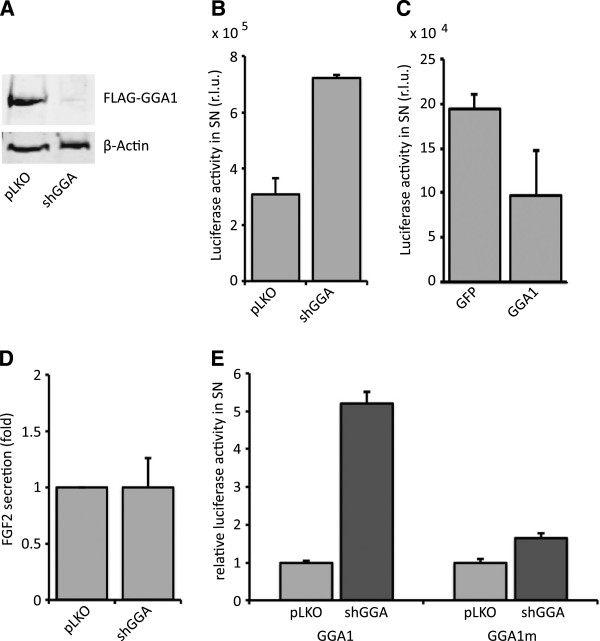
**Release of Gaussia luciferase is suppressed by GGA1. A**, To test efficiency of shRNA mediated GGA knockdown 293 T cells expressing Flag-tagged GGA1 were co-transfected with pLKO control vector or pLKO shGGA. Cell lysates were resolved on 10% PAGE and transferred to PVDF membranes. The blots were probed with anti-Flag M2 or anti-β-actin antibody. **B**, 293 T cells were co-transfected with dNGLUC and the shRNA vectors pLKO (control) or pLKO shGGA and cultured for 72 h. Supernatants were harvested and analyzed for Gaussia luciferase activity using a PerkinElmer TopCount. **C**, 293 T cells were co-transfected with dNGLUC and an expression vector for GFP or GGA1 and cultured for 24 h prior to analysis of GLUC activity in supernatants as in **A**. **D**, FGF2 was transfected into 293 T cells together with a control vector (pLKO) or an shRNA vector targeting GGA1. FGF2 secretion into supernatants was measured using an ELISA kit (R&D Systems) after 24 h. E, 293 T cells expressing dNGLUC were co-transfected with pLKO vector control or shGGA and wild-type Flag-GGA1 or a mutant version of GGA1 that is refractory to shRNA mediated knockdown by shGGA (GGA1m). Supernatants were harvested after 48 h and analysed for GLUC activity as previously described. **B**-**E**, shown are the results from three independent experiments plus standard deviation. R.L.U. – relative light units, SN - supernatant.

In order to identify proteins that regulate the non-conventional secretion of GLUC, shRNA-mediated knockdown of candidate proteins involved in Golgi trafficking was examined. shRNA-mediated knockdown of a Golgi-associated protein, Golgin-Gamma-ear-Adaptin (GGA)1 (Figure 6A) was found to significantly enhance release of dNGLUC (Figure 6B). Furthermore, overexpression of GGA1 resulted in a strong reduction of dNGLUC release (Figure 6C). As a control, the release of another unconventionally secreted protein, Fibroblast Growth Factor (FGF)-2, was investigated. Release of FGF2 from 293 T cells was not affected by shRNA-mediated knockdown of GGA1 (Figure 6D), in accordance with the notion that FGF2 secretion is insensitive to treatment with Brefeldin A and requires a plasma membrane transporter [40]. Enhanced release of dNGLUC upon shRNA-mediated knockdown of GGA1 could be reversed by transfection of a Flag-tagged GGA1 construct refractory to knockdown (GGA1m; Figure 6E). We therefore conclude that GGA1 is an endogenous inhibitor of dNGLUC release.

## Discussion

*Gaussia* luciferase activity can be released from cells by three distinct mechanisms: 1) classical N-terminal signal peptide mediated secretion (GLUC); 2) internal signal peptide mediated release(GFP-_SP_GLUC and actin-_SP_GLUC); and 3) non-conventional release in the absence of a signal peptide (dNGLUC). While assays based on conventional secretion of Gaussia luciferase have been previously described [41], the data presented here support two novel applications of Gaussia luciferase: (1) with the use of an internal signal peptide to monitor bicistronic gene expression and secretion and (2) use as a reporter to study non-conventional secretion mechanisms.

One advantage of secreted luciferases is their potential for deployment in multiplexed reporter assays. However, one limitation for multiplexing is the availability of expression cassettes for multiple reporter genes. To circumvent this problem, bicistronic expression systems are available such as those based on internal ribosomal entry sites (IRES) or self-cleaving peptides (e.g. the picornavirus 2A sequence [42]). However, IRES-based systems can result in inefficient expression of the downstream gene, and P2A self-cleaving peptides leave both a C-terminal extension on the upstream gene product, and an N-terminal proline on the downstream product. It has been shown that in the presence of an N-terminal signal peptide at the front of two sequential proteins, an internal signal sequence can function to export the two proteins from the cell [27]. The internal signal sequence can function either as a transfer signal resulting in export of the downstream peptide or alternatively as a stop-transfer signal resulting in membrane anchoring of the full peptide sequence. The bicistronic expression system proposed here based on GFP fused to Gaussia luciferase results in efficient export of the peptide downstream of the signal peptide. Interestingly, the Gaussia luciferase internal signal sequence functions in the absence of an N-terminal signal sequence as demonstrated for GFP-_SP_GLUC and β-actin-_SP_GLUC. A systematic comparison of signal sequence strength has previously shown that the Gaussia luciferase signal peptide is one of the most efficient secretory signals known [43], which may explain the effective export of proteins even when the signal sequence is placed between two reporter proteins. Our results indicate GLUC should have utility for bicistronic reporter gene expression and secretion.

Protein secretion is a complex process that involves intra-cellular trafficking steps and post-translational modifications to the secretory cargo. Canonical secretion involves translocation of the nascent polypeptide from the ribosome to the lumenal endoplasmatic reticulum (ER) followed by vesicular transport through the Golgi. This type of secretion is initiated by interaction of a hydrophobic N-terminal signal sequence on the polypeptide with the signal recognition particle. Some proteins, however, lack an N-terminal signal peptide and are secreted by a non-conventional secretion that is insensitive to treatment with inhibitors of ER/Golgi trafficking such as Brefeldin A [44,45]. Proteins known to undergo non-conventional secretion include interleukin (IL) -1α and -1β, fibroblast growth factor (FGF) 1 and 2, HIV tat, transglutaminases and galectins, among others. In some cases, including those of HIV tat, FGF-2 and cellular transglutaminase, the export is constitutive, whereas in other cases secretion is initiated by a regulated event, such as the cleavage of interleukins 1α and 1β by caspase-1. Multiple mechanisms for non-conventional secretion have been identified, including lysosomal secretion, plasma membrane shedding, exosomal release, as well as secretion through plasma membrane resident transporters [46]. In general, non-conventional secretion is poorly understood and should benefit from the application of novel tools that permit the use of unbiased approaches such as high-throughput screening technologies. We present here evidence that dNGLUC can be used as reporter gene to study non-conventional secretion.

Recently, an involvement of autophagy in non-conventional secretion has been described in yeast, *Drosophila*, mammals and plants [28-31,47]. We have shown that cellular stress responses and autophagy are not involved in dNGLUC release, because the same amount of dNGLUC was released from ATG5-/- null cells as was released from control cells. In addition, treatment with autophagy inducers or inhibitors had no effect on release of dNGLUC. The underlying mechanisms of this type of secretion are worthy of further investigation, firstly because the process is unusual and may expose new aspects of general cell biology, and secondly because such an understanding would enhance the development of reporter systems based on this type of secretion.

In this study we have also shown that GGA1 suppresses release of dNGLUC from cells. GGAs are a family of monomeric clathrin adaptors that mediate the sorting of transmembrane cargo from the trans-Golgi network to late endosomes [48]. GGA1 can directly interact with clathrin [49-51], AP1 [52,53], ARF [54], Rabaptin-5 [55,56] and with a variety of cargo molecules [57], and is required for sorting of the lysosomal targeting receptor, mannose-6-phosphate receptor [48,52]. It has also been observed that overexpression of GGA3 inhibits retrovirus assembly [58], and that the molecule interacts with TSG101, an ubiquitin-binding component of the multivesicular body that is a key regulator of HIV budding. In addition, GGAs have been implicated in trafficking and processing of APP [59-62] have been found to bind to β-secretase [44,63,64] and appear to restrain Aβ secretion, as indicated by the enhanced secretion observed following siRNA mediated knockdown of GGA1. Here, we have observed suppression of secretion of dNGLUC by GGA1.

In principle GGA1 might suppress release of dNGLUC by interfering with lysosomal sorting. In this scenario dNGLUC might be sorted in large measure to the lysosome and consequently, suppression of lysosomal targeting would result in a net increase of flux through other trafficking routes. However, lysosomal inhibitors such as chloroquine had no effect on release of dNGLUC, diminishing the attractiveness of this hypothesis.

## Conclusions

In summary, we present here a novel type of secretion that occurs in the absence of a signal peptide, can ferry diverse cargo to the extracellular space, is enhanced by growth factor stimulation and does not require autophagy. In the future, it will be desirable to address the molecular mechanisms of this type of secretion in more detail. The secreted luciferase presented here should be an excellent tool to expose additional features of the mechanism using high-throughput screening technologies.

## Methods

### Plasmids and constructs

pEAK12-dNGLUC, GFP-dNGLUC and Actin-LC3-dNGLUC have been described previously. GFP-_SP_GLUC and Actin-_SP_GLUC were generated by inserting the wild-type GLUC sequence into pEAK12-GFP and pEAK12-Actin-LC3-dNGLUC between Eco*RI* and Not*I* restriction sites. GGA1 was amplified by PCR using primers 5’-GACGAATTCATGGAGCCCGCGATGG-3’ and 5’- GACGCGGCCGCCTAGAGGCTACCCCAGG-3’ and inserted via Eco*RI*/Not*I* into pEAK13-Flag. A GGA1 mutant that is refractory to siRNA treatment was generated by overlap extension PCR using the following internal primers: cctccaAgAccTaaAaatgtgA and ccacatttttaggtcttggagg. pLKO shRNA vectors were obtained from SigmaAldrich. The sequence for knockdown of GGA1 was: CGGCCGAAGAATGTGATCTTTGAA.

### Cell culture

293 T and COS-7 cells were cultured in Dulbecco’s modified essential media (DMEM, Gibco) supplemented with 10% fetal bovine serum (Sigma-Aldrich), 1% GlutaMAX (Gibco) and 1% penicillin/streptomycin (Gibco). Doxycycline-inducible ATG5-/- murine embryonic fibroblasts (MEF) [39] were obtained from Dr. Noboru Mizushima (Tokyo Dental Institute) and were cultured in DMEM (Gibco) supplemented with 1% GlutaMAX, 1% penicillin/streptomycin, 1% sodium pyruvate (PAA) and 10% FBS (Sigma-Aldrich). Cells were induced with 1 μg/ml doxycycline (SigmaAldrich) for the indicated time points.

### Small molecule compound treatment

All compounds were obtained from Sigma-Aldrich and dissolved in DMSO as 10 mM stock solutions unless otherwise indicated. Brefeldin A was purchased as a solution (10 mg/ml) from Sigma-Aldrich. Chloroquine (Sigma-Aldrich) was kept as a 25 mM stock solution in water. 293 T cells expressing dNGLUC were washed once with culture medium before addition of compounds in complete DMEM. Concentrations and time of incubation are detailed in the Figure legends. Epidermal Growth Factor (EGF) was obtained from PeproTech. COS-7 cells were starved overnight in 0.1% serum and stimulated with 100 ng/ml EGF at 37°C.

### Transfection and retroviral transduction

293 T cells were transfected with expression plasmids using calcium phosphate precipitation as described [65] or Lipofectamine 2000 (Invitrogen) at 200 ng/well in a 96-well plate. DNA was mixed with 0.5 ml Lipofectamine in 50 mL serum-free DMEM and incubated for 30 min at room temperature before addition to cells.

To express dNGLUC in MEFs, pMOWSdSV-dNGLUC was used for retroviral transduction. Retroviral stocks were prepared by transient transfection of 293 T cells. A solution consisting of 1 mL DMEM, 75 μL of 1 mg/mL polyethylenimine, 5 μg of each of the viral helper plasmids VSV-G and Gag Pol and 20 μg of pMOWSdSV-dNGLUC was prepared, incubated for 20 minutes and added to a 10 cm plate of 293 T cells at 60% confluency. The following day the culture medium was replaced with 7 mL of fresh culture medium. The resulting virus-containing supernatant was collected two days after transfection and filtered through a 0.45 μm cellulose acetate filter. Upon addition of polybrene to a final concentration of 1 μg/mL, the virus solution was added to a 6 cm plate of MEFs at 70% confluency. After 24 h the solution was exchanged with regular culture media.

### Protein concentration

293 T cells were cultured in serum-free medium overnight, supernatants were collected, filtered through 0.45 μm filter and 500 μl portions were loaded onto Amicon Ultra spin prep columns (Millipore) with a cut-off of 3000 Da. Samples were spun at 14,000 rpm and 4°C for 60 min prior to elution of concentrated protein samples. Samples were then mixed with sample buffer and analyzed by immuno-blotting. The volume was concentrated ~10-fold.

### Luciferase assay

Native Coelenterazine (Biotium) was dissolved at a concentration of 1 mg/mL in acidified Methanol. An assay buffer was prepared by mixing the coelenterazine solution 1:100 with Gaussia luciferase buffer (0.1% disodium phosphate, 5% glycerol, 150 mM sodium bromide, 1 mM EDTA, 25 mM Tris–HCl pH8 and 2 mM ascorbic acid). 10 μL of supernatants were transferred to black 96-well plates and mixed with 90 μL assay buffer prior to analysis of luciferase activity in a PerkinElmer Envision II plate photometer.

### Cell viability assay

An assay solution was prepared by diluting Cell Counting Kit-8 stock solution (Sigma) 1:50 in PBS. Culture medium was aspirated from 293 T cells in 96 well plates and replaced with 100 μL of the assay solution. After 2 h incubation at 37°C the absorbance at 450 nm was measured with a PerkinElmer Envision II.

#### Elisa

Fibroblast Growth Factor 2 (FGF2) in supernatants was measured with a R&D Systems ELISA kit according to the manufacturer’s instructions.

### Immuno-blotting

293 T cells were lysed in NP40 lysis buffer (50 mM Tris–HCl pH8, 0.5% NP40, 0.1% EDTA, 10% glycerol and protease inhibitors), mixed with 2× SDS sample buffer and loaded onto 10% PAGE pre-cast gels (BioRad). Proteins were transferred to a PVDF membrane and subsequently blocked for 1 to 2 h in blocking solution (5% skimmed milk and 0.1% Tween in PBS). Proteins of interest were probed with primary antibodies overnight at 4°C in blocking solution. HRP-conjugated secondary antibody incubation was conducted for 1 h at room temperature in blocking solution. Antibody bound proteins were detected using ECL reagents. The following antibodies were used: anti-GLUC (Ketteler et al. [19,20], anti-Flag M2 (Sigma-Aldrich), anti-β-Actin (Abcam), anti-LC3 (Sigma-Aldrich), anti-ATG5 (Nanotools), HRP anti-mouse (Santa-Cruz) and HRP anti-rabbit (Santa-Cruz).

## Abbreviations

SP: Signal peptide; SN: Supernatant; GLUC: Gaussia luciferase; dNGLUC: N-terminal deletion mutant of Gaussia luciferase; GGA1: Golgi-associated Gamma adaptin ear containing ARF binding protein 1; GFP: Green fluorescent protein; LC3: MAP1LC3B.

## Competing interests

The authors declare that they have no competing interests.

## Authors’ contributions

CL performed experiments and helped to draft the manuscript. JF performed and designed experiments. DE, NAT, JH and IL performed experiments. JKV helped with statistical analysis and design of experiments. BS and RK conceived the study and designed experiments. RK performed experiments and wrote the manuscript. All authors read and approved the final manuscript.
